# Correction: Proteolysis targeting chimeras (PROTACs) come of age: entering the third decade of targeted protein degradation

**DOI:** 10.1039/d5cb90053k

**Published:** 2026-01-05

**Authors:** Michael J. Bond, Craig M. Crews

**Affiliations:** a Department of Pharmacology, Yale University New Haven CT 06511 USA craig.crews@yale.edu; b Department of Molecular, Cellular, and Developmental Biology, Yale University New Haven CT 06511 USA; c Department of Chemistry, Yale University New Haven CT 06511 USA

## Abstract

Correction for ‘Proteolysis targeting chimeras (PROTACs) come of age: entering the third decade of targeted protein degradation’ by Michael J. Bond *et al.*, *RSC Chem. Biol.*, 2021, **2**, 725–742, https://doi.org/10.1039/D1CB00011J.

The authors regret that an incorrect version of Fig. 4 was included in the original article which labelled Bordoxalone as an *Irreversible* covalent E3 ligase recruiting element (E3RE), when Bordoxalone should have been labelled as a *Reversible* covalent E3RE. This was stated correctly in the main text. The correct version of Fig. 4 is presented below.

**Fig. 4:**
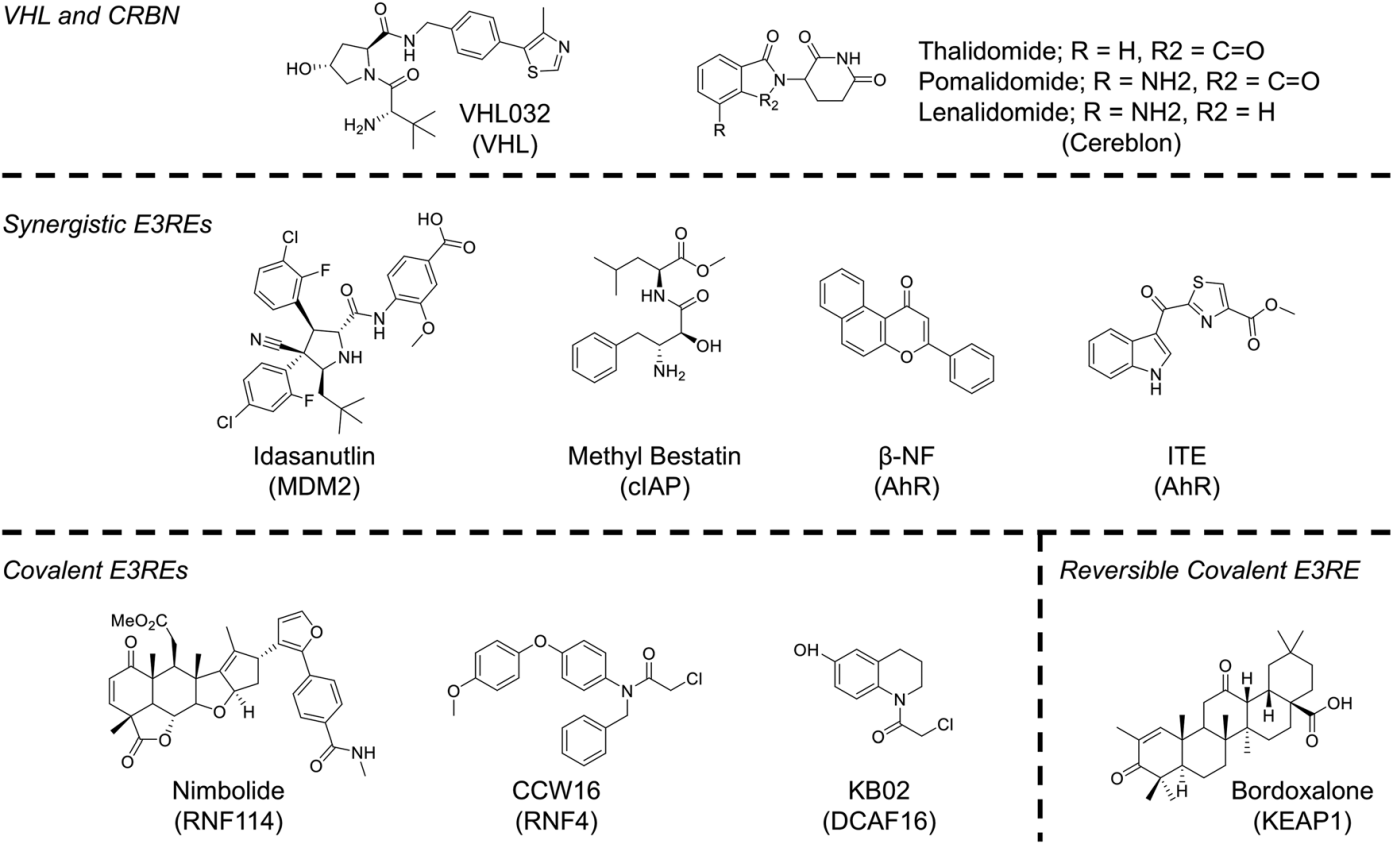
Chemical structures of E3 ligase recruiting elements (E3REs) used in TPD.

The Royal Society of Chemistry apologises for these errors and any consequent inconvenience to authors and readers.

